# Assessing the potential of improving growth and survival to the eyed stage in selectively bred Arctic charr (*Salvelinus alpinus*)

**DOI:** 10.1111/jbg.12509

**Published:** 2020-10-03

**Authors:** Christos Palaiokostas, Henrik Jeuthe, Dirk‐Jan De Koning

**Affiliations:** ^1^ Department of Animal Breeding and Genetics Swedish University of Agricultural Sciences Uppsala Sweden; ^2^ Aquaculture Center North Kälarne Sweden

**Keywords:** Arctic charr, egg survival, genotype‐by‐environment interaction, selective breeding

## Abstract

The Arctic charr breeding programme has been a main driving force for developing the aquaculture industry in Sweden. Selection has been performed for almost 40 years using animals from a closed breeding nucleus. The aim of the current study was to evaluate the potential of further improving growth‐related traits taking into account the existence of genotype‐by‐environment interaction. Furthermore, we investigated the magnitude of the genetic component associated with survival to the eyed stage and potential associations with inbreeding coefficients. A preliminary heritability estimate of 0.23 (*SE* 0.20) was obtained for survival to the eyed stage using records spanning from 2000 to 2017 (*n* = 230). Moreover, moderate‐to‐high heritability estimates (0.27–0.49) were obtained for growth‐related traits (body weight and length), using animals from the latest generation of selection (year class 2017). Those animals (*n* = 2,776), originating from 55 full‐sib families, were split into two groups and reared in separate land‐based facilities of commercial fish farms in Sweden. The growth‐related traits were recorded twice in both sites when animals were of >1 and >2 years of age. Existence of sexual growth dimorphism was indicated with the males having on average 6%–8% higher total length and 22%–34% higher body weight. Furthermore, high genetic correlations regarding growth traits were obtained amongst animals reared at the two different sites (0.82–0.95). In addition, we assessed the accuracy of best linear unbiased prediction (BLUP)‐derived estimated breeding values (EBVs) when phenotypes from each rearing site were subsequently masked and used as a validation set. A mean prediction accuracy of 0.60 (length) and 0.64 (weight) were derived for both rearing sites. Overall, our results suggest that further growth improvements should be possible in the subsequent generations of selection. Finally, even though indications for the existence of an underlying genetic component(s) involved in survival to the eyed stage were obtained additional data will be required for elucidating its magnitude.

## 1 INTRODUCTION

Arctic charr (*Salvelinus alpinus*) farming in Sweden is a promising industry, despite the relatively small production volume (1,310 tn; SCB, 2017). A national breeding programme has been running for approximately 40 years resulting in an improved strain capable of reaching market size (>600 g) one year earlier compared to wild stocks (Eriksson et al., 2010) with an estimated genetic gain of 10%–11% per generation (Carlberg et al., 2018). A closed breeding nucleus originating from lake Hornavan in Sweden has been maintained since the beginning of the programme during the 1980s.

Typically, the Arctic charr breeding programme has been based on 45–125 full‐sib families (Nilsson et al., 2010) reared in separate tanks until a size of 30–60 g upon which the breeding candidates are marked using passive integrated transponder (PIT) tags and reared communally thereafter. Selection has been conducted on discrete generations with an approximate interval of 4 years. Additionally, the genetically improved material has been disseminated over the years to various farms across the country. Identification of the most promising selection candidates is practised by estimating breeding values (EBVs) for growth‐related traits (body weight and length) through the best linear unbiased prediction (BLUP) methodology (Henderson, 1975), while at the same time striving to minimize inbreeding accumulation through avoiding crossing together full or half sibs.

Prior studies have reported moderate‐to‐high heritabilities for growth‐related traits ranging between 0.34 and 0.52 at the first generations of the breeding programme (Elvingson & Nilsson, 1994; Nilsson, 1992). However, a more recent study focusing on animals from the 2009 year class obtained a wide range of heritability estimates (0.01–0.47) for different life stages (Nilsson et al., 2016). Furthermore, since the main aim of the breeding programme was to disseminate the improved genetic material and promote Arctic charr farming across the country, the magnitude of genotype‐by‐environment (GxE) interaction is a factor of paramount importance. Existence of significant GxE can potentially result in a re‐ranking of EBVs depending on the rearing environment, jeopardizing the efficiency of selection. Currently, EBVs are calculated using information from the breeding nucleus site only. Prior studies on GxE interaction for growth traits in Arctic charr conducted on the year classes 1993 and 2009 have estimated GxE of differing magnitude (Nilsson et al., 2010, 2016) indicating that the efficiency of selection could differ amongst farming sites.

In contrast to the substantial improvement regarding growth‐related traits, survival to the eyed stage in Arctic charr is remarkably lower compared to other salmonids (Jeuthe et al., 2013, 2016). It is worth mentioning that the eyed stage in salmonids is considered a critical developmental stage prior to which the eggs are highly sensitive to any type of handling. Most importantly, while survival to the aforementioned stage in farmed salmonids is usually above 90% (Vandeputte et al., 2002; Vehviläinen et al., 2010), the available recordings point towards a significant drop of successfully fertilized Arctic charr eggs which in certain occasions can be lower than 30% (personal communication). A substantial variation in terms of successful hatching rates (0%–97%) amongst the recorded single pair crosses suggests that underlying genetic components might be of particular significance (Jeuthe et al., 2013).

Currently, no information exists regarding the magnitude of the genetic component associated with survival to the eyed stage in Arctic charr. Furthermore, no prior study has focused on obtaining accuracy estimates regarding the efficiency of BLUP‐derived EBVs for growth‐related traits in Arctic charr reared in different sites. The aim of the current study was to investigate the magnitude of genetic components involved in survival of Arctic charr embryos to the eyed stage and the potential for further improving growth‐related traits.

A heritability estimate of survival to the eyed stage was obtained using available individual family recordings from 2000 to 2017. Moreover, updated estimates of heritability on growth‐related traits for the latest year class of the breeding programme (2017) were used as indicators of the genetic potential for further improvement. Additionally, genetic correlations regarding growth traits (body weight and length) were estimated amongst animals from the 2017 year class reared in two different environments. Finally, we attempted to quantify the efficiency of BLUP‐derived EBVs for growth traits on animals reared at different environments. In particular, phenotypic information from each rearing environment was subsequently masked and used as a validation set.

## 2 MATERIALS AND METHODS

### 2.1 Breeding population

The breeding nucleus is located at the facilities of Aquaculture Center North (ACN) in Kälarne, Jämtland, Sweden. Pedigree data were available for eight generations of selectively bred Arctic charr (*n* = 21,221). Full‐sib families were obtained through artificial stripping involving single pair matings. Overall, the breeding design involved separately fertilizing the eggs of two dams from one sire. The Arctic charr spawning season in the breeding nucleus facility occurs in October–November with the set‐up crosses usually occurring within an overall timeframe of 3 weeks. Eggs from each full‐sib family were incubated in individual trays until hatching. To minimize age differences between the different spawning events, egg incubation was synchronized to the same degree days using heated water (6°C). Unfertilized and dead eggs were removed daily from the incubation trays to avoid the spread of bacterial and fungal infections. Following completion to the eyed stage, live and dead eggs were sorted and counted. Survival to the eyed stage was estimated for each family by dividing the number of eyed eggs with the total number of stripped eggs. Overall, egg survival data up to the eyed stage were available for individual crosses spanning between 2000 and 2017.

### 2.2 Growth recordings—Year class 2017

Eggs from the latest year class of the Arctic charr breeding programme hatched during February 2017 with the fry from each full‐sib family being transferred to individual 1 m^3^ tanks. As such, a partly confounding effect occurs between the additive genetic effect and the common environmental component due to rearing individual families in separate tanks. Overall, the 2017 year class was comprised of 55 full‐sib families (40 sires and 55 dams). The animals were tagged using PIT at an average weight of 60 g and split into two groups with equal family representation and reared communally thereafter. The size of each group ranged between 4 and 30 animals for each family (mean = 26, *SD* = 4). The first group (Site A, *n* = 1,469) remained in Kälarne (breeding nucleus) for the entire duration of the study, while the other group (Site B, *n* = 1,307) was transferred to Timrå, Västernorrland County, in June 2018. Site A used lake water of ambient temperature, while site Site B used groundwater of fairly constant temperature (6–8°C).

Growth measurements involving body weight and total length were taken twice from each individual fish during 2018 and 2019 when the animals were between 580 and 940 posthatching days. More specifically, measurements for animals of Site A were taken on 25–26 September 2018 and on 18–20 September 2019. Animals from Site B were measured on 1–2 October 2018 and on 27–28 August 2019. On all occasions, body weight and length were recorded to the closest gram and millimetre, respectively. Finally, during the reproductive season of 2019 (October–November) phenotypic sex was recorded from the >2‐year‐old animals assigning them to males, females or immature.

### 2.3 Investigating for association between inbreeding coefficients and survival to the eyed stage

Inbreeding coefficients for all pedigree‐recorded animals were estimated using the INBUPGF90 software from the BLUPF90 suite (Aguilar & Misztal, 2008). Existence of associations between the inbreeding coefficient and survival to the eyed stage were investigated with both estimating the Pearson correlation coefficient and through applying linear regression of the survival to the eyed stage on the inbreeding coefficients of the parental fish. In the latter case a linear model was fitted as follows:

(1) y=Xb+e,

where **y** is the vector of recorded survival data regarding the eyed stage (ranging between 0–0.98) and **b** is the vector of fixed effects. The fixed effects included age, year class, the inbreeding coefficient, and the weight of the parental fish. **X** is the incidence matrix relating phenotypes with the fixed effects. Finally, **e** is the vector of residuals ~ $N\;(0,\;I\sigma_{e}^{2})$ and $\sigma_{e}^{2}$ is the residual variance.

### 2.4 Heritability estimation for growth traits and egg survival

Heritability estimates were obtained for body weight, length, and survival to the eyed stage. The corresponding variance components were estimated using AIREMLF90 (Misztal et al., 2018) using the following single‐trait animal models for the survival to the eyed stage and the growth‐related traits, respectively:

(2) y=Xb+Zu+e,

(3) y=Xb+Zu+Tc+e,

where **y** is the vector of recorded phenotypes and **b** is the vector of fixed effects. The **y** vector in the case of egg survival contains the individual family survival ratio at eyed stage. In the case of growth‐related traits, the fixed effects included age, tank and rearing site, while in the case of egg survival the fixed effects included age, year class and the weight of dam and sire. For the latter trait, available data spanned across different year classes and included broodfish of varying age. Moreover, broodfish weight was added as covariate due to prior indications in the literature studying fertility traits in farmed fish (Su et al., 2016). X is the incidence matrix relating phenotypes with the fixed effects, and Z is the incidence matrix relating phenotypes with the random animal effects. u is the vector of random animal effects ~ $N\;(0,\;A\sigma_{g}^{2})$ with A corresponding to the additive relationship matrix, and $\sigma_{g}^{2}$ is the additive genetic variance. T corresponds to the incidence matrix relating phenotypes with the common full‐sib effect, $c∼\;N\;(0,\;I\sigma_{\text{FS}}^{2})$ is the vector of common random environmental effects due to the initial rearing of each full‐sib family in separate tanks until tagging and $\sigma_{\text{FS}}^{2}$ is the corresponding variance. Finally, e is the vector of residuals ~ $N\;(0,\;I\sigma_{e}^{2})$ and $\sigma_{e}^{2}$ the residual variance.

Heritability for survival to the eyed stage was obtained using the following formula:

$$h^{2}\;=\;\frac{\sigma_{g}^{2}}{\sigma_{g}^{2}\;+\;\sigma_{e}^{2}}$$

while heritability estimates for the growth‐related traits were obtained as follows:

$$h^{2}\;=\;\frac{\sigma_{g}^{2}}{\sigma_{g}^{2}\;+\;\sigma_{\text{FS}}^{2}\;+\;\sigma_{e}^{2}}$$

In the case of model (3), the common full‐sib effect was estimated using the following formula:

$$c^{2}\;=\;\frac{\sigma_{\text{FS}}^{2}}{\sigma_{g}^{2}\;+\;\sigma_{\text{FS}}^{2}\;+\;\sigma_{e}^{2}}$$

### 2.5 Genetic correlations among the growth‐related traits

Bivariate models with the same fixed and random effect structure as in (2) were used in order to estimate genetic correlations between the recorded growth traits either in >1 or >2‐year‐old animals (2017 year class) separately or between the different life stages. Furthermore, the aforementioned models were applied to estimate genetic correlations for the growth traits amongst the two rearing sites in order to gain insight regarding the magnitude of GxE interactions. The models had the following format:

(4) $$\left[\begin{array}{c} y_{1}\\ y_{2} \end{array}\right]\;=\;\left[\begin{array}{cc} X_{1} & 0\\ 0 & X_{2} \end{array}\right]\;\left[\begin{array}{c} b_{1}\\ b_{2} \end{array}\right]\;+\;\left[\begin{array}{cc} Z_{1} & 0\\ 0 & Z_{2} \end{array}\right]\;\left[\begin{array}{c} u_{1}\\ u_{2} \end{array}\right]\;+\;\left[\begin{array}{c} e_{1}\\ e_{2} \end{array}\right],$$

where **y_i_** the vectors of the phenotypic traits under study. **b_i_,** $u_{i}\;∼\;N\;(0,\;G_{0}\;⊗\;A)$ and $e_{i}\;∼\;N\;(0,\;R_{0}\;⊗\;I)$ the vectors of fixed, random effects and residuals respectively. G_0_ and R_0_ the 2x2 variance–covariance matrices for random effects and residuals, while ⊗ represents the Kronecker product. X_i_ and **Z**_**i**_ were the corresponding design matrices for the fixed and random effects as previously described.

The genetic correlations (*r*_xy_) amongst traits or sites were calculated according to:

$$r_{i,j}\;=\;\frac{\sigma_{\alpha i,\;\alpha j}}{\sqrt{\sigma_{\alpha i}^{2}\;\times \;\sigma_{\alpha j}^{2}}}$$

where, $\sigma_{\alpha i,\alpha j}$ corresponds to the genetic covariance variance between the traits or sites in the bivariate model and $\sigma_{\alpha i}^{2},\;\sigma_{\alpha jj}^{2}$ correspond to the additive genetic variances of the different traits or sites.

### 2.6 Estimation and accuracy of breeding values across rearing sites

Breeding values for body weight and length were obtained using the BLUPF90 software suite (Misztal et al., 2018). The breeding values were obtained both for model (2) and for the model containing the random common full‐sib environmental effect (3).

Moreover, a cross‐validation scheme was performed in order to test prediction accuracy of the EBVs between the different rearing sites. In particular, the phenotypic value (total weight or length) of each rearing site was sequentially masked and breeding values were estimated using information from the other site. The above was performed when the animals were >2 year of age (2019 recordings). The accuracy of the EBVs for the rearing site in question was approximated as follows:

$$\text{accuracy}\;=\;\text{correlation}\;(\mathrm{EBV},\;\hat{y})/h,$$

where $\hat{y}$ is the vector of body weight or length adjusted for the fixed effects, **EBV** is the vector of obtained breeding values and h is the square root of the heritability. Finally, the bias of the EBVs was inferred based on the slope of the regression of the adjusted phenotype for fixed effects on the EBV.

## 3 RESULTS

### 3.1 Descriptive metrics for the survival to the eyed stage data

**FIGURE 1:**
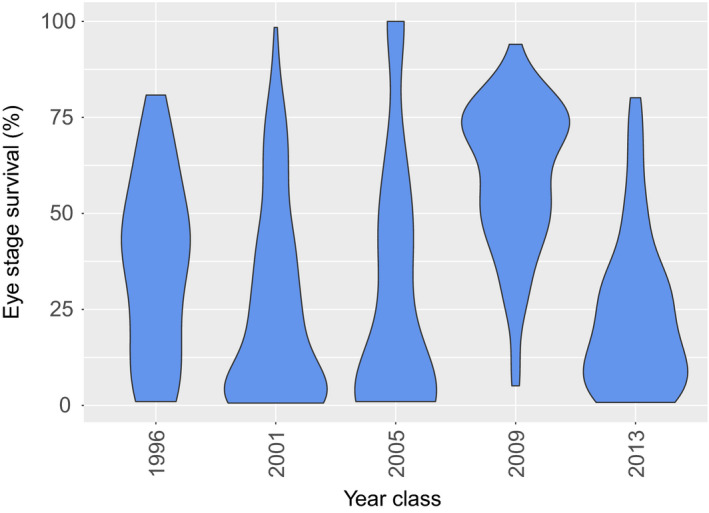
Percentage of eyed stage egg survival per year class

Eyed stage survival data from 524 records were available. The data set was comprised of 5 year classes, with the age of the broodstock ranging between 3 and 6 years. In total, 455 dams and 323 sires were used. The weight of the dams ranged between 1,363 and 7,329 g, while the weight of sires ranged between 452 and 7,775 g. Eyed stage survival per individual mating ranged between 0% and 98% (Figure 1).

### 3.2 Inbreeding coefficients—heritability for egg survival

**FIGURE 2:**
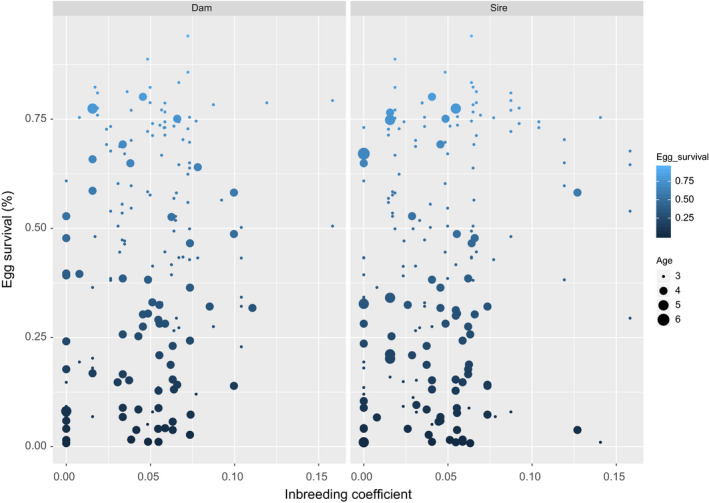
Inbreeding coefficients and egg survival percentage for dams and sires

The mean inbreeding coefficient across the recorded year classes was 5.8 (%) (Table 1). Out of the 524 available records of egg survival data, only 230 were recorded in the pedigree. As such, investigating for associations between the inbreeding coefficients and survival to the eyed stage and heritability estimation was performed using the records containing pedigree information. The inbreeding coefficients for animals with available recordings for survival to the eyed stage ranged between 0 and 0.16. The Pearson correlation coefficient between inbreeding coefficient and eyed stage egg survival was 0.17 for the dams and 0.15 for the sires (Figure 2). Moreover, the obtained regression coefficient of survival to the eyed stage on the inbreeding coefficient of the parental fish was not significant (*p* = .23). Finally, the obtained heritability estimate for survival to the eye stage was 0.23 (*SE* 0.20).

**TABLE 1:** Average inbreeding (%) over year classes 1996–2017

Year class	No sires	No dams	Average inbreeding (%)
1996	22	47	1.53
2001	33	48	3.73
2005	40	49	4.12
2009	88	123	5.31
2013	35	50	6.12
2017	40	55	5.62

### 3.3 Descriptive metrics of recorded traits for the 2017 year class

**FIGURE 3:**
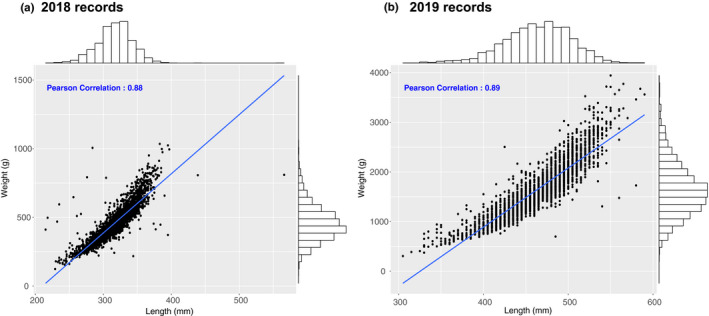
Correlation between body weight and total length amongst 2018 and 2019 recordings

Growth‐related recordings regarding body weight and total length were obtained from 2,776 year class 2017 Arctic charr on two occasions when A) the animals were >1 and B) >2 years of age. In the 2018 recordings, the mean weight and length were 460 g and 317 mm, respectively, while in the 2019 measurements the mean weight was 1,648 g and the total length 464 mm (Table 2). The Pearson correlation between the phenotypic recordings of weight and length was 0.88 and 0.89, respectively (Figure 3). Amongst animals with recorded sex (*n* = 1,256), 48% were females and 52% males. Phenotypic males had higher mean body weight and length during both the 2018 and 2019 recordings. In particular, males had on average 6%–8% higher total length and 22%–34% higher body weight (Table 3).

**TABLE 2:** Mean body weight and total length for recorded on two occasions for year class 2017

Site	Weight 1st (g)	Weight 2nd (g)	Length 1st (mm)	Length 2nd (mm)
A	441	1,439	315	450
B	482	1,882	319	479

**TABLE 3:** Mean growth‐related recordings per sex

Sex	Weight 1st (g)^(^ *^SE^* ^)^	Weight 2nd (g)^(^ *^SE^* ^)^	Length 1st (mm)^(^ *^SE^* ^)^	Length 2nd (mm)^(^ *^SE^* ^)^
Female	423^(4.3)^	1,342^(14.3)^	311^(1.02)^	440^(1.35)^
Male	516^(4.8)^	1,798^(19.8)^	330^(0.96)^	478^(1.28)^
Immature	451^(3.4)^	1,706^(14.2)^	314^(0.66)^	467^(1.06)^

Note

Superscripts correspond to standard errors

### 3.4 Heritability estimates–genetic correlations for growth traits

Heritability estimates for the measured growth traits without including the common full‐sib effect ranged between 0.28 and 0.44. The highest heritability was found for body weight during the 2018 recordings. Reduced heritability estimates were obtained when we used the model with the common full‐sib effect. Using model (3), the obtained heritability estimates for length and weight ranged between 0.27–0.28 and 0.33–0.37, respectively. The common full‐sib effect for the 2018 recordings was 0.03 (*SE* 0.06) and 0.05 (*SE* 0.05) for weight and length, respectively, while the corresponding values for the 2019 recordings were <0.01. The values of the corresponding standard errors under model (2) more than doubled in the case of model (3).

The genetic correlations among the recorded growth traits ranged between 0.66 and 0.88. Genetic correlations between the 2018 and 2019 recordings were 0.80 (*SE* 0.06) and 0.81 (*SE* 0.07) for weight and length, respectively. The lowest genetic correlation was between body weight of 2019 and total length of the 2018 recordings (Table 4).

**TABLE 4:** Heritability (diagonal), genetic correlations (upper diagonal) and corresponding standard errors (superscripts) for growth‐related traits

	Weight 1st^(^ *^SE^* ^)^	Weight 2nd ^(^ *^SE^* ^)^	Length 1st ^(^ *^SE^* ^)^	Length 2nd^(^ *^SE^* ^)^
No common full‐sib effect				
Weight 1st ^(^ *^SE^* ^)^	0.44^(0.08)^	0.81^(0.07)^	0.88^(0.04)^	0.52^(0.12)^
Weight 2nd ^(^ *^SE^* ^)^	–	0.33^(0.06)^	0.66^(0.10)^	0.78^(0.07)^
Length 1st^(^ *^SE^* ^)^	–	–	0.40^(0.07)^	0.80^(0.06)^
Length 2nd ^(^ *^SE^* ^)^	–	–	–	0.28^(0.06)^
Common full‐sib effect				
Weight 1st ^(^ *^SE^* ^)^	0.37^(0.17)^	N/A^a^	N/A^a^	N/A^a^
Weight 2nd ^(^ *^SE^* ^)^	–	0.33^(0.06)^	N/A^a^	N/A^a^
Length 1st^(^ *^SE^* ^)^	–	–	0.27^(0.14)^	N/A^a^
Length 2nd ^(^ *^SE^* ^)^	–	–	–	0.33^(0.06)^

^a^ No convergence of model was obtained.

### 3.5 Genetic correlations amongst the two rearing sites

The common full‐sib effect was not included due to convergence issues. Regarding the 2018 recordings, genetic correlations of weight and length amongst the two rearing sites were 0.83 (*SE* 0.08) and 0.95 (*SE* 0.05). During the phenotypic recordings of 2019, the genetic correlations for weight and length for the two rearing sites were 0.91 (*SE* 0.07) and 0.82 (*SE* 0.10), respectively.

### 3.6 Family ranking and obtained accuracies

**FIGURE 4:**
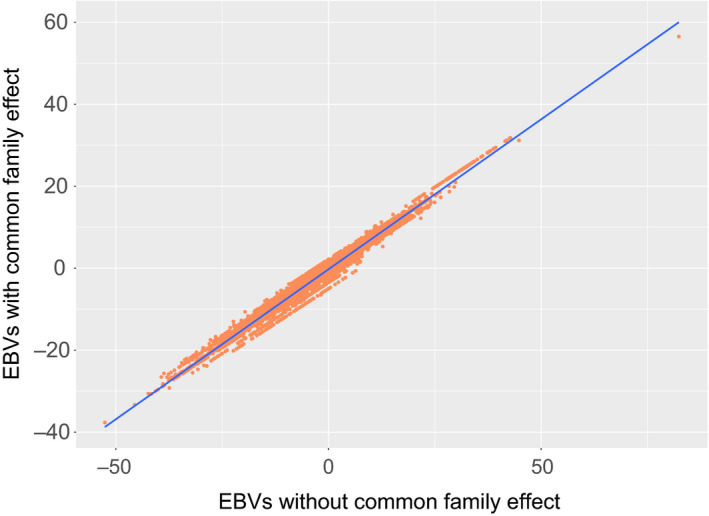
EBVs regarding length estimated with or without inclusion of a random family effect to account for systematic bias due to rearing each family in a different tank until PIT tagging

**FIGURE 5:**
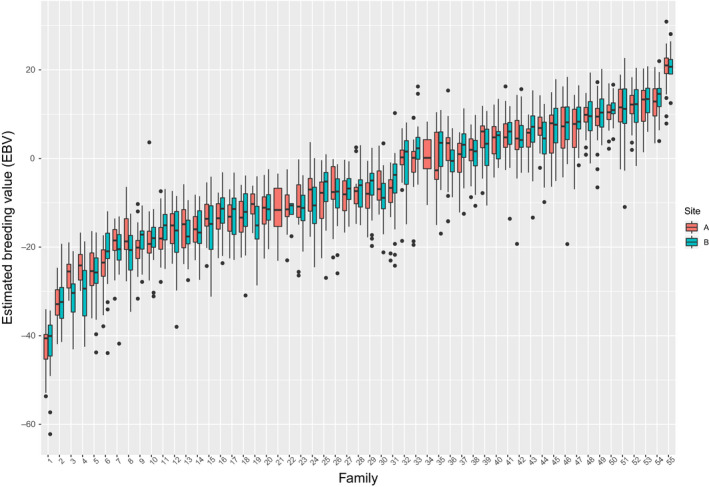
Estimated breeding values regarding length of each full‐sib family for each rearing site

Estimated breeding values were obtained using both models (2) and (3). The Pearson correlation coefficient between the above was found to be 0.99 for both weight and length (Figure 4). Family ranking based on BLUP‐derived EBVs using either model (2) or (3) was in general consistent amongst the two rearing sites (Figure 5). Furthermore, the mean accuracy of the EBVs when the phenotypic information from each rearing site was subsequently masked and used as a validation set was 0.60 and 0.64 for length and weight, respectively. The obtained accuracy in the case of animals reared at Site A was 0.62 (length) and 0.67 (weight) while in the case of animals from Site B the accuracy estimates were 0.58 (length) and 0.61 (weight). The corresponding bias estimates for Site A were 1.001 (length) and 1.479 (weight), while in the case of Site B bias was 0.730 (length) and 0.576 (weight).

## 4 DISCUSSION

### 4.1 Reproductive success—survival to the eyed stage

Interestingly, a reduction in a wide range of reproductive traits is particularly evident in aquaculture species compared to wild stocks (Farquharson, Hogg, & Grueber, 2018). Low reproductive success is a major issue that hinders the expansion of Arctic charr farming. Both genetic and environmental factors with most pronounced the rearing temperature are suspected to be implicated (Olk et al., 2019). The available recordings regarding survival to the eyed stage suggested a different pattern for year class 2009 with a higher survival rate (Figure 1). Interestingly, a new water inlet was installed in 2010 resulting in reducing substantially the water temperature during the summer months (Jeuthe et al., 2015). Nevertheless, subsequent reproductive seasons did not show a consistent improvement. It is worth mentioning that low survival to the eyed stage in Arctic charr is also observed in farms using water of low temperature (6–8°C) throughout the year (personal communication).

A moderate heritability estimate of 0.23 was obtained for survival to the eyed stage indicating that genetic factors can influence substantially the reproductive success. However, the aforementioned was accompanied by a high standard error of 0.20. According to literature, heritability estimates for early‐life survival in salmonids are skewed towards lower values (median value of 0.06; Carlson & Seamons, 2008). Prior studies pointed towards a significant dam effect for eyed stage survival in both Atlantic salmon and rainbow trout (Rye, Lillevik, & Gjerde, 1990; Vandeputte et al., 2002). Accumulation of inbreeding due to the small effective population number that is frequently encountered in aquaculture farming (as is likely the case with the current study) could be a major factor explaining the deterioration of reproductive traits leading to low egg survival. However, in our study a low correlation (dams = 0.17; sires = 0.15) and a non‐significant regression coefficient were found between the estimated inbreeding coefficients and the eyed stage egg survival. Nevertheless, due to the small sample size (230 records) of our study we have limited margin on drawing solid inferences regarding the magnitude of the genetic component associated with eyed stage egg survival in Arctic charr.

It is worth emphasizing that the Swedish Arctic charr breeding programme is selecting primarily for increased growth. Research in various species demonstrated that selecting solely for growth can negatively impact reproductive related traits (Narinc et al., 2014). However, the above is not consistent and there are cases were selecting for growth does not negatively affect reproductive traits (Santana et al., 2012). Unfortunately, in the current study we were not able to draw conclusions regarding the existence of a potentially negative effect of selecting for increased growth in the survival to the eyed stage due to limited number of available recordings. It would be of primary importance though to investigate closer the latter in the future reproductive seasons.

### 4.2 Sexual dimorphism in growth‐related traits

To the best of our knowledge no prior study indicated the existence of sexual growth dimorphism in Arctic charr. Even though, we were not able to decipher the phenotypic sex from all the animals of the 2017 year class, clear differences regarding growth amongst the two sexes were suggested by our data (*n* = 1,256). Interestingly, growth differences were apparent well in advance of sexual maturation with the males having on average 6%–8% higher total length and 22%–34% higher body weight over the two‐time recordings. The above implies that taking into account the phenotypic sex when EBVs is of high importance from a management/planning perspective as well. The usage of genetic markers would be a most valuable solution which would allow to decipher sex at an early age well in advance of any selection decisions. Sexual growth dimorphism has been recently suggested also in the case of Norwegian Atlantic salmon with the males appearing 2.8%–8.7% heavier amongst the different time spanning recordings (Thorland et al., 2020). Most interestingly, a reverse situation appears to exist in the case of North American Atlantic salmon with the females at least during the freshwater stage appearing heavier (Schaeffer et al., 2018).

### 4.3 Genetic parameters for growth traits

Moderate‐to‐high heritability estimates (depending on used model) were obtained in our study for growth‐related traits (body weight and length) suggesting that further cumulative improvement is feasible in the subsequent generations of the Arctic charr breeding programme. Typically, heritability estimates for growth traits in salmonids range between 0.10 and 0.60 (Elvingson & Nilsson, 1994; Kause et al., 2007; Nilsson, 1992; Tsai et al., 2015; Yáñez et al., 2016). The Arctic charr breeding programme has been running for approximately 40 years, being currently at the 8th generation of selection. From the start of the breeding programme, selection has been performed including only animals from the breeding nucleus without any introduction of external genetic material. As such, investigating the potential for further improvements regarding growth is of paramount importance.

On both recordings of our study moderate heritability estimates were obtained for the growth traits. Furthermore, the genetic correlations between the different time points were above 0.80 when concerning the same trait. Contradicting evidence has been documented in the literature regarding the correlation between growth traits recorded at different life stages in fish (Vandeputte et al., 2008). Most strikingly, a genetic correlation of only 0.10 (*SE* 0.17) was recently found between weight recordings 8 months apart in Atlantic salmon (Thorland et al., 2020), while on the other hand a genetic correlation between fingerling and harvest weight of 0.77 was estimated in channel catfish (Bosworth et al., 2020).

### 4.4 Architecture of genetic models in aquaculture selective breeding designs

Not surprisingly, the actual architecture of the utilized statistical model for estimating the relevant variance components can artificially lead to either over‐ or underestimation of the heritability component. Particularly in the case of farmed fish, pedigree recording is more complicated as opposed to livestock due to the inherent nature of the aquaculture activity. The typical salmonid breeding design involves rearing animals from the same full‐sib family in individual tanks until a size suitable for PIT tagging (Gjedrem, 2010; Gjedrem & Robinson, 2014). Thereafter, the breeding candidates can be communally reared. The above design can, if not properly accounted for, lead to overestimated heritability estimates (Gallardo et al., 2010). Especially in scenarios where the time difference between communal and separate family rearing is small, exacerbated heritability values can be obtained (Martínez et al., 1999; Sae‐Lim et al., 2013).

Nevertheless, heritability estimation through models containing a random common full‐sib effect aiming to correct for the communal rearing period can lead to undesired convergence problems of numerical nature and difficult to interpret results due to confounding with the animal additive effects. The above situation is particularly profound in cases where multi‐trait models are used. The inclusion of a common full‐sib effect in those cases can lead to poor convergence of the estimated parameters or capturing all the variance of the trait. In connection to the results of our study, several cases have been documented in the literature where the estimation of genetic correlations in multi‐trait models did not include the common full‐sib effect to avoid non‐convergence issues (Maluwa et al., 2006; Sae‐Lim et al., 2013). Additionally, the inclusion of the common full‐sib effect results in a considerable increase in the accompanying standard errors highlighting the confounding effect with the random animal component. A more than twofold increase in the relevant standard errors was observed in our study when the common full‐sib effect was included. Similar observations were documented in prior studies (Sae‐Lim et al., 2013).

Overall, a careful experimental design, where similar numbers of hatched larvae from each family are introduced to all individual tanks and the time interval of rearing each family to separate tanks is reduced to the absolute necessary without jeopardizing the animals health, due to the unavoidable stress caused by PIT tagging, would alleviate the mentioned issues regarding heritability estimation. In our study, PIT tagging was conducted when the animals reached a mean average weight of 60 g which is towards the upper limit of the usual size of tagging for salmonids. The above could be a reason for obtaining high heritabilities for growth traits which where towards the upper limit of recorded values in the literature. Nevertheless, regardless of the used models, heritability estimates above 0.27 for both length and weight were obtained indicating that a robust growth improvement should be feasible in the subsequent generations of selected Arctic charr. Furthermore, the estimated common full‐sib effect from the univariate models was limited to 0.05 or lower for the 2018 recordings, while the corresponding estimates for the 2019 recordings were below 0.01. Finally, indistinguishable BLUP‐derived EBVs were obtained in our study using either models (*r* = .99) providing reassurance that at least in this case the common full‐sib effect would not influence substantially the ranking of the selection candidates.

### 4.5 Genotype‐by‐environment interaction

GxE can potentially pose insurmountable hurdles in any breeding programme. Particularly, sensitive to GxE are designs similar to the Arctic charr breeding programme where EBVs are solely estimated using records from breeding candidates reared at the nucleus facilities. High GxE can result in EBV re‐ranking depending on rearing environment jeopardizing the selection efficiency. In high extremes, the only solution could be setting different breeding lines accustomed for each rearing environment. However, that would require high financial costs and, especially in cases of small production volumes as in Arctic charr, would be deemed economically infeasible. In aquaculture species, a mean genetic correlation (.72) across rearing environments has been documented indicating that a moderate re‐ranking of EBVs could occur (Sae‐Lim et al., 2016). Genetic correlations of.82 and above were obtained for the recorded growth traits amongst animals reared in the two different sites indicating that GxE would not have a considerable impact on the efficiency of selection in this case. The above appears to be in accordance with a prior study where weak GxE has been indicated (Nilsson et al., 2016). Nevertheless, we need to acknowledge the fact that all recordings in our study took place in land‐based facilities. Since rearing in lake cages from juvenile stage to harvest size is the most commonly farmed method, it would be of high value to investigate the magnitude of GxE in the aforementioned environment.

### 4.6 Accuracy of EBVs

Genetic correlation estimates can provide a solid clue regarding the efficiency of selection across environments. In the current study, the above was reinforced when a cross‐validation scheme was performed where prediction accuracies were obtained for each subsequent rearing site using information only from the other. An overall mean accuracy of 0.6 and 0.64 was estimated across the rearing sites for length and weight, respectively. To our knowledge, no accuracy values of BLUP‐derived EBVs have been recorded across rearing environments in any aquaculture species. Taking into account accuracy estimates from prior aquaculture studies (Houston et al., 2020), the estimated accuracy in our study could be deemed satisfactory. Nevertheless, differences in the estimated bias were obtained depending on the used trait and site of rearing. In particular, in the case of length less bias (Site A: 1.001, Site B:0.730) was observed compared to weight (Site A: 1.480, Site B: 0.576). Focusing on length, while prediction for the animals on site Site A resulted in minute bias, when the same design was applied for the animals on site Site B a considerable bias was obtained implying that the EBVs on the latter occasion were inflated. Interestingly, animals from site Site B experienced fairly stable water rearing temperature due to well‐supplied water. Therefore, when the training set was comprised of the Site B animals the environmental noise due to temperature fluctuations was minimized resulting in the estimation of relatively unbiased EBVs. Nevertheless, in the case of weight substantial bias was obtained for both sites which could indicate that other factors could also play a role.

## 5 CONCLUSION

Overall, moderate‐to‐high heritability estimates for growth‐related traits were obtained, indicating that further growth improvements would be feasible in subsequent generations. Only a preliminary heritability estimate of eyed stage egg survival was obtained due to the small sample size suggesting though that selection practices could be of value. High genetic correlations and prediction accuracy for the growth‐related traits were estimated supporting the current breeding scheme of a single nucleus. Further improvements could be realized through utilizing genomic technologies. Genotyping by sequencing approaches could offer an economically attractive approach that could lead to substantial accuracy improvements regarding the estimation of breeding values (Palaiokostas et al., 2020). Especially, if the above is coupled with single‐step BLUP methodologies (Aguilar et al., 2011; Garcia et al., 2018; Legarra et al., 2014; Misztal et al., 2020) where only a subset of the selection candidates is genotyped a most effective utilization of the available economic resources could be achieved.

## CONFLICT OF INTEREST

The authors declare that they have no conflicting interests.

## AUTHOR CONTRIBUTIONS

CP, HJ and DK conceived the study and contributed to designing the experimental structure. HJ carried the data recordings. CP carried out data analysis and breeding value estimations. All authors contributed to drafting the manuscript.

## ETHICAL APPROVAL

No experimental crossings were performed for the needs of the current study. Instead, available data recordings from the national Swedish breeding programme of Arctic charr were utilized.

## Data Availability

The data sets used in this study were from a commercial breeding programme. Data are available upon request from the corresponding author.
